# Implications of pleiotropy: challenges and opportunities for mining Big Data in biomedicine

**DOI:** 10.3389/fgene.2015.00229

**Published:** 2015-06-30

**Authors:** Can Yang, Cong Li, Qian Wang, Dongjun Chung, Hongyu Zhao

**Affiliations:** ^1^Department of Mathematics, Hong Kong Baptist UniversityHong Kong, Hong Kong; ^2^Hong Kong Baptist University Institute of Research and Continuing EducationShenzhen, China; ^3^Program in Computational Biology and Bioinformatics, Yale UniversityNew Haven, CT, USA; ^4^Department of Public Health Sciences, Medical University of South CarolinaCharleston, SC, USA; ^5^Department of Biostatistics, Yale School of Public HealthNew Haven, CT, USA; ^6^Department of Genetics, Yale School of MedicineNew Haven, CT, USA; ^7^VA Cooperative Studies Program Coordinating CenterWest Haven, CT, USA

**Keywords:** genome-wide association studies (GWAS), pleiotropy, functional annotation, mining Big Data in biomedicine, data integration

## Abstract

Pleiotropy arises when a locus influences multiple traits. Rich GWAS findings of various traits in the past decade reveal many examples of this phenomenon, suggesting the wide existence of pleiotropic effects. What underlies this phenomenon is the biological connection among seemingly unrelated traits/diseases. Characterizing the molecular mechanisms of pleiotropy not only helps to explain the relationship between diseases, but may also contribute to novel insights concerning the pathological mechanism of each specific disease, leading to better disease prevention, diagnosis and treatment. However, most pleiotropic effects remain elusive because their functional roles have not been systematically examined. A systematic investigation requires availability of qualified measurements at multilayered biological processes (e.g., transcription and translation). The rise of Big Data in biomedicine, such as high-quality multi-omics data, biomedical imaging data and electronic medical records of patients, offers us an unprecedented opportunity to investigate pleiotropy. There will be a great need of computationally efficient and statistically rigorous methods for integrative analysis of these Big Data in biomedicine. In this review, we outline many opportunities and challenges in methodology developments for systematic analysis of pleiotropy, and highlight its implications on disease prevention, diagnosis and treatment.

## 1. Introduction

In the past decade, genome-wide association studies (GWAS) have been conducted to study the genetic basis for thousands of phenotypes (Hindorff et al., 2009; Eicher et al., 2015), including diseases (e.g., the seven diseases from WTCCC, The Wellcome Trust Case Control Consortium, 2007), clinical traits (e.g., cholesterol levels), anthropometric traits (e.g., height, Wood et al., 2014), brain structures (Hibar et al., 2015) and social behaviors (e.g., educational attainment, Rietveld et al., 2013; marriage, Domingue et al., 2014). As of April, 2015, more than 15,000 single-nucleotide polymorphisms (SNPs) have been reported to be significantly associated (*p* <5 × 10^−8^) with at least one phenotype (see GWAS catalog, Welter et al., 2014). By exploring these fruitful findings from GWAS, recent progress has suggested that a single locus may influence multiple seemingly different phenotypes (Solovieff et al., 2013). This phenomenon, termed “Pleiotropy,” was formally introduced into the scientific literature by the German geneticist Ludwig Plate in 1910 (Stearns, 2010). Accumulating evidence suggests that pleiotropy widely exists among complex traits, such as psychiatric disorders (Cross-Disorder Group of the Psychiatric Genomics Consortium, 2013a,b), metabolic syndrome traits (Vattikuti et al., 2012) and cancers (Sakoda et al., 2013). Examples of pleiotropy in human diseases include the *PTPN22* locus associated with multiple auto-immune disorders (Cotsapas et al., 2011), such as rheumatoid arthritis, Crohn's disease, and type I diabetes; the *TERT-CLPTM1L* locus associated with bladder, glioma, and lung cancers (Fletcher and Houlston, 2010). Although the first survey (Sivakumaran et al., 2011) of pleiotropic effects (PE) was published in 2011, it may underestimate PE as the database of GWAS results was far less than what is available today. Fortunately, the Genome-Wide Repository of Associations between SNPs and Phenotypes (GRASP) database has been built up as a repository of many results from published GWAS (Leslie et al., 2014). A recent update (Eicher et al., 2015) on GRASP has provided even more comprehensive GWAS results—about 8.87 million SNP-phenotype associations in 2082 studies with *p* ≤0.05. Such a rich data resource allows characterizing the molecular mechanisms of PE on diverse phenotypes. Undoubtedly, it will not only greatly deepen our understanding of the genetic architecture that underlies complex human phenotypes, but also have clinically important implications.

## 2. Benefits from characterization of pleiotropic effects

To demonstrate the potential benefits from characterization of PE, we consider a recent study from Butte's team at Stanford (Li et al., 2014b). Based on available information from the VARiants Informing MEDicine (VARIMED) database (Ashley et al., 2010), Butte's team hypothesized that diseases (e.g., type 2 diabetes) and non-disease traits (e.g., blood pressure and cholesterol levels; for convenience, we shall refer to “non-disease traits” as “traits” hereafter) could be related to each other through shared genetic variants. They first identified significant associations between 801 unique genes and 69 diseases, and between 796 unique genes and 85 traits. Next, they identified 120 disease-trait pairs that could be reliably linked via shared genetic variants, and 26 of them are novel to the community. Among these novel findings, five pairs can be directly validated by electronic medical records of patients from three independent clinical centers: Stanford Hospital and Clinics, Mount Sinai Medical Center and Columbia University Medical Center. For example, gastric cancer and the serum magnesium level is one of these five pairs. This pair is linked via three genes—*MUC1*, *THBS3*, and *TRIM46*, as implicated by some previous studies of gastric cancer (Wadhwa et al., 2013) and the serum magnesium level (Meyer et al., 2010). To validate this disease-trait pair, 804 patients were selected as cases because they had a magnesium measurement 1 year before their diagnosis of gastric cancer, and 324,160 individuals who had at least one magnesium measurement without diagnosis of gastric cancer were selected as controls. The comparison showed that the cases had a significantly higher magnesium levels than the controls. If this finding could be further replicated independently, it would have a very important clinical implication—the serum magnesium level could be used as a bio-marker which predicts the risk of gastric cancer 1 year beforehand.

There would be more benefits in clinical practice if the biological mechanisms of PE on some disease-trait pairs could be timely validated. One immediate benefit is the development of more affordable clinical tests, such as blood tests and imaging tests, for disease prevention and diagnosis, as implied by the real example above. Another potential benefit is the discovery of new drugs for disease treatment. Consider two diseases that are connected through a common biological mechanism. If a drug works well in treating one disease, it will also likely to be effective for the other disease. For example, calcium antagonist drugs have been used for the treatment of hypertension since 1960s (Wood et al., 1999). Recently, the Psychiatric Genomics Consortium (PGC) identified the L-type calcium channel subunit gene *CACNA1C* as a risk gene for several psychiatric disorders (Cross-Disorder Group of the Psychiatric Genomics Consortium, 2013b). As reported in Solovieff et al. (2013), this new finding has drawn the attention in trials of calcium antagonist drugs because these drugs could be potentially useful for psychiatric disorder treatment.

## 3. Statistical perspectives on characterization of pleiotropic effects

Although results from thousands of GWAS are readily available, systematical analysis of existing GWAS results toward fully characterizing PE is not trivial. Historically, the genome-wide significant level was set to *p* ≤ 5 × 10^−8^ (McCarthy et al., 2008) as empirical data in European-decent GWAS suggested adjustment for 1–2 million independent tests (The International HapMap Consortium, 2005; McCarthy et al., 2008). However, it may not be wise to narrow down the search range of PE within the significant GWAS hits because they can only explain a very small proportion of phenotypic variance, which is known as the “missing heritability” phenomenon (Manolio et al., 2009). In 2010, Yang et al. showed that 45% of the variance for human height of 3925 unrelated individuals could be explained by 294,831 common SNPs (Yang et al., 2010). So far, researchers have found similar results for many other complex phenotypes (Visscher et al., 2012), such as metabolic syndrome traits (Vattikuti et al., 2012), and psychiatric disorders (Cross-Disorder Group of the Psychiatric Genomics Consortium, 2013a; Yang et al., 2014). An important lesson learned from GWAS is the polygenic architecture of complex human phenotypes—besides the significant GWAS hits, a complex human phenotype is often affected by many genetic variants with small or moderate effects (Visscher et al., 2012). Due to the limited sample size, these genetic risk variants may not be identified at the stringent genome-wide significant level, i.e., *p* ≤ 5 × 10^−8^. The survey of PE in 2011 showed that 4.6% of identified SNPs and 16.9% of identified genes were associated with multiple phenotypes (Sivakumaran et al., 2011). This result could be an underestimate of PE as many variants with weak or moderate effects have not been identified. For better characterization of PE among multiple phenotypes, the uncertainties arise in single-GWAS analysis should be taken into account. For example, it may be not easy to determine whether a variant with *p*-value about 10^−5^ is disease-associated or not if we only focus on the variant itself. Statistics plays a critical role in incorporating indirect but relevant information (Efron, 2010) to account for uncertainties as discussed below.

Before discussion of statistical methods to characterize PE, we first introduce the local and global measures of PE. Originally, the term “pleiotropy” referred to the phenomenon that a single locus affects two or more phenotypes (Stearns, 2010). Let *u*^(1)^ and *u*^(2)^ be the effect sizes of this locus on two phenotypes, respectively. According to this definition, this locus is said to have pleiotropic effect if both *u*^(1)^ and *u*^(2)^ are nonzero. Clearly, this is a local measure of pleiotropy as it only refers to a specific locus. In the genomics era, the local measure has been extended to a global one which is defined as the correlation between the effect sizes of all genetic variants on the two phenotypes (Cross-Disorder Group of the Psychiatric Genomics Consortium, 2013a). Here we first introduce the method to estimate the global PE, and then discuss the issue about localization of PE in the next section.

At first glance, it seems to be straightforward to obtain the global PE by a two-step strategy: estimate the effect sizes individually for each variant and then calculate their correlation. However, accurate estimation of all the effect sizes in the first step can be very challenging due to limited sample sizes. Uncertainty arises in characterizing global PE due to the estimation error accumulated in the first step, resulting in the reduction of efficiency in correlation estimation in the second step. Fortunately, linear mixed models (LMM) arise as a powerful tool to address this challenge (Lee et al., 2012).

For simplicity, let us consider GWAS of two distinct phenotypes and without overlaped samples. Denote the phenotypes and genotypes as **y**^(*k*)^ ∈ ℝ^*n*_*k*_ × 1^ and **G**^(*k*)^ ∈ ℝ^*n*_*k*_ × *M*^, respectively, where *M* is the number of SNPs in the genotyped matrix and *n_k_* is the sample size of the *k*-th GWAS, *k* = 1, 2. Bivariate LMM can be used for estimating global PE as follows:

$$\begin{array}{l} y^{\left(1\right)}=X^{\left(1\right)}\beta^{\left(1\right)}+G^{\left(1\right)}u^{\left(1\right)}+e^{\left(1\right)},\\ y^{\left(2\right)}=X^{\left(2\right)}\beta^{\left(2\right)}+G^{\left(2\right)}u^{\left(2\right)}+e^{\left(2\right)}, \end{array}$$

where **X**^(*k*)^ is a design matrix of fixed effects collecting all the covariates (e.g., sex and age) for the *k*-th phenotype, **β**^(*k*)^ is the coefficient vector of the fixed effects, **u**^(*k*)^ is the coefficient vector of the genetic effects viewed as random, and **e**^(*k*)^ is the residual, *k* = 1, 2. Let *u*^(1)^_*m*_ and *u*^(2)^_*m*_ be the *m*-th element of **u**^(1)^ and **u**^(2)^, respectively. Then they are assumed to jointly follow the bivariate Gaussian distribution as

$$\left[\begin{array}{c} u_{m}^{\left(1\right)}\\ u_{m}^{\left(2\right)} \end{array}\right]\sim N\left(\left[\begin{array}{c} 0\\ 0 \end{array}\right],\left[\begin{array}{cc} \sigma_{u_{1}}^{2} & \rho_{u}\sigma_{u_{1}}\sigma_{u_{2}}\\ \rho_{u}\sigma_{u_{1}}\sigma_{u_{2}} & \sigma_{u_{2}}^{2} \end{array}\right]\right)\text{​},$$

where σ*_u_k__* is the standard deviation of the effect sizes on the *k*-th phenotype, and ρ*_u_* ∈ [−1, 1] is the genetic correlation to capture global PE. In such a way, these random effects **u**^(*k*)^ can be integrated out analytically, which helps us bypass the great challenge in accurate estimation of weak individual effects. After that, the model parameters (σ_*u*_1__, σ_*u*_2__, ρ_*u*_) can be estimated using maximum likelihood (ML) or restricted maximum likelihood (REML) (Lee et al., 2012). In other words, all available information can be simultaneously incorporated under a statistically rigorous framework unlike the naive two-step strategy discussed above. Through this approach, the genetic relationship between five psychiatric disorders has been explored based on genome-wide SNPs (Cross-Disorder Group of the Psychiatric Genomics Consortium, 2013a). The estimated genetic correlation was very strong between schizophrenia and bipolar disorder (${\hat{\rho }}_{u}$ = 0.68 with s.e. 0.04), low between schizophrenia and autism spectrum disorders (${\hat{\rho }}_{u}$ = 0.16 with s.e. 0.06), and non-significant for some other pairs of disorders. The results may have clinically important implications for the classification of psychiatric disorders which has been done conventionally on the basis of symptoms. Due to the limited space here, readers interested in technical details of LMM shall refer to Jiang (2007) and McCulloch et al. (2008).

As discussed above, the global PE can be estimated accurately after accounting for the uncertainties in estimating individual genetic effects. Similarly, leveraging pleiotropy and accounting for this estimation uncertainty can lead to significant improvement of disease risk prediction, as recently demonstrated in Li et al. (2014a); Maier et al. (2015). Interested readers can easily try the LMM approach in GWAS data analysis using efficient softwares, e.g., GCTA (Yang et al., 2011) and GEMMA (Zhou and Stephens, 2014).

## 4. Challenges and opportunities in characterization of pleiotropic effects

Although LMM is a very powerful tool to capture the global PE, systematical localization of PE is still at the beginning stage. In this section, we outline the challenges and opportunities from the statistical point of view.

There are two major challenges in localization of PE. First, a substantial proportion of phenotypic variance is attributed to many variants with small effects due to the polygenicity of complex human phenotypes. Identification of those risk variants with a very high certainty may not be supported by the sample size of current GWAS. A loose threshold (e.g., *p* ≤ 0.05) may lead to many false positives (Type I error), while a stringent threshold (e.g., *p* ≤ 5 × 10^−8^) may produce too many false negatives (type II errors). Therefore, it may be problematic to examine pleiotropy based on the intersection of identified GWAS loci after a simple thresholding. Second, spurious pleiotropy may appear due to the artifact in experimental design and the linkage disequilibrium (LD) among genetic variants. On one hand, ascertainment bias can introduce spurious PE when sample recruitment based on the first phenotype changes the prevalence of the second phenotypes (Smoller et al., 2000). For example, it is possible that patients who suffer from two or more illnesses are more likely to be recruited than those with only one. As accumulation of experience in GWAS design, the sampling strategy and techniques to control confounding factors have been improved (Feero et al., 2010). Thus, this type of artifact may be reduced over time (Solovieff et al., 2013). On the other hand, the strong LD with two risk variants may cause spurious PE. Suppose SNPs A and B are two nearby risk variants for two distinct phenotypes. It is possible that they are strongly linked due to LD but locate in two genes with different functions. Statistically, it would be very challenging to distinguish this spurious PE from truly biological PE, given the limited information. Incorporation of functional information of the two SNPs can be very helpful to exclude this spurious PE as their distinct functions tell their differences.

To systematically characterize PE, additional information should be incorporated into statistically rigorous analysis. Instead of solely relying on genetic information, the Encyclopedia of DNA Elements (ENCODE) project (The ENCODE Project Consortium, 2012) has provided a comprehensive map of the regulatory elements in the human genome. Although most of its results are from cell lines, it has highlighted the importance of regulatory regions in the human genome. Another project, named Roadmap Epigenomics project (Kundaje et al., 2015), used primary cells instead of cell lines, aiming at providing reference epigenomes of more than one hundred tissues and cell types to tackle human diseases. Apart from epigenetic markers, the Genotype-Tissue Expression project (GTEx) (Lonsdale et al., 2013) aims at collection of 20,000 tissues from 900 donors, serving as a comprehensive atlas of gene expression and regulation across human tissues. Clearly, integration of pleiotropy and functional annotation to drive advanced scientific hypotheses is calling for rigorous analysis (Ritchie et al., 2015). Besides the statistical methods mentioned in the timely review paper by Solovieff et al. (2013), several statistical approaches to characterizing PE have been developed more recently, including GPA (Chung et al., 2014), CPASSOC (Zhu et al., 2015b), MGAS (Van der Sluis et al., 2015), Bayesian Test for Colocalization (Giambartolomei et al., 2014) and others.

As an example, here we briefly introduce GPA (Chung et al., 2014), a statistical approach recently developed by us. As a first attempt, GPA is designed to integrate pleiotropy and functional annotation information for risk SNP prioritization, and significance assessment of pleiotropy and annotation enrichment. A notable feature of GPA is that it only requires the summary statistics from GWAS, rather than the genotype data at the individual level, as its input, which greatly facilitates GWAS data integration. GPA has been applied to analyze psychiatric disorders and bladder cancer, with various types of functional annotation. Our analysis results suggest that integration of genomics data may potentially lead to abundant novel findings (Chung et al., 2014).

It is noteworthy that characterization of PE should not be restricted to phenotypes at the organismal level. Investigation the PE between the organismal phenotype (e.g., disease status) and the cellular phenotype (e.g., DNA methylation, gene expression, protein expression and metabolite abundance) may lead to even more fruitful discoveries as the genetic variants often have much larger effect sizes on cellular phenotypes (Battle et al., 2015). Mining the biomedical data representing different biological processes at different layers is becoming feasible as these data have been well organized recently, including the GTEx database (Lonsdale et al., 2013), a cross-platform collection of human gene expression data (Zhu et al., 2015a), a tissue-based human protein database (Uhlén et al., 2015) and an atlas of genetic influences on human blood metabolites (Shin et al., 2014). The potential impact of PE at different layers can be amplified as the costs of cellular trait measurements continue to drop with the advent of new technologies.

## 5. Conclusion

The advent of big data has revolutionized biomedical research. We are able to comprehensively characterize the health condition of a human subject with quantitative measurements generated at both cellular and organismal levels. From these data, we may find direct or indirect evidence to resolve long-standing problems and motivate advanced scientific hypotheses. Characterization of PE based on integration of various data types is such an exciting process, while the risk of identification of spurious PE may be largely increased due to the enlarged search space. We believe that statistically rigorous methods which effectively account for uncertainties in data integration will continue to play a critical role in improving statistical power and decreasing false positive findings.

### Conflict of interest statement

The authors declare that the research was conducted in the absence of any commercial or financial relationships that could be construed as a potential conflict of interest.

## References

[B1] Ashley E. A. Butte A. J. Wheeler M. T. Chen R. Klein T. E. Dewey F. E.. (2010). Clinical assessment incorporating a personal genome. Lancet 375, 1525–1535. 10.1016/S0140-6736(10)60452-7 20435227 PMC2937184

[B2] Battle A. Khan Z. Wang S. H. Mitrano A. Ford M. J. Pritchard J. K.. (2015). Impact of regulatory variation from RNA to protein. Science 347, 664–667. 10.1126/science.1260793 25657249 PMC4507520

[B3] Chung D. Yang C. Li C. Gelernter J. Zhao H. (2014). GPA: a statistical approach to prioritizing GWAS results by integrating pleiotropy and annotation. PLoS Genet. 10:e1004787. 10.1371/journal.pgen.1004787 25393678 PMC4230845

[B4] Cotsapas C. Voight B. F. Rossin E. Lage K. Neale B. M. Wallace C.. (2011). Pervasive sharing of genetic effects in autoimmune disease. PLoS Genet. 7:e1002254. 10.1371/journal.pgen.1002254 21852963 PMC3154137

[B5] Cross-Disorder Group of the Psychiatric Genomics Consortium. (2013a). Genetic relationship between five psychiatric disorders estimated from genome-wide SNPs. Nat. Genet. 45, 984–994. 10.1038/ng.2711 23933821 PMC3800159

[B6] Cross-Disorder Group of the Psychiatric Genomics Consortium. (2013b). Identification of risk loci with shared effects on five major psychiatric disorders: a genome-wide analysis. Lancet 381, 1371–1379. 10.1016/S0140-6736(12)62129-1 23453885 PMC3714010

[B7] Domingue B. W. Fletcher J. Conley D. Boardman J. D. (2014). Genetic and educational assortative mating among us adults. Proc. Natl. Acad. Sci. U.S.A. 111, 7996–8000. 10.1073/pnas.1321426111 24843128 PMC4050565

[B8] Efron B. (2010). The future of indirect evidence. Stat. Sci. 25, 145–157. 10.1214/09-STS308 21243111 PMC3019763

[B9] Eicher J. D. Landowski C. Stackhouse B. Sloan A. Chen W. Jensen N.. (2015). GRASP v2. 0: an update on the genome-wide repository of associations between SNPs and phenotypes. Nucleic Acids Res. 43, D799–D804. 10.1093/nar/gku1202 25428361 PMC4383982

[B10] Feero W. G. Guttmacher A. E. Manolio T. A. (2010). Genomewide association studies and assessment of the risk of disease. New Engl. J. Med. 363, 166–176. 10.1056/NEJMra0905980 20647212

[B11] Fletcher O. Houlston R. S. (2010). Architecture of inherited susceptibility to common cancer. Nat. Rev. Cancer 10, 353–361. 10.1038/nrc2840 20414203

[B12] Giambartolomei C. Vukcevic D. Schadt E. E. Franke L. Hingorani A. D. Wallace C.. (2014). Bayesian test for colocalisation between pairs of genetic association studies using summary statistics. PLoS Genet. 10:e1004383. 10.1371/journal.pgen.1004383 24830394 PMC4022491

[B13] Hibar D. P. Stein J. L. Renteria M. E. Arias-Vasquez A. Desrivières S. Jahanshad N.. (2015). Common genetic variants influence human subcortical brain structures. Nature 520, 224–229. 10.1038/nature14101 25607358 PMC4393366

[B14] Hindorff L. A. Sethupathy P. Junkins H. A. Ramos E. M. Mehta J. P. Collins F. S.. (2009). Potential etiologic and functional implications of genome-wide association loci for human diseases and traits. Proc. Natl. Acad. Sci. U.S.A. 106, 9362–9367. 10.1073/pnas.0903103106 19474294 PMC2687147

[B15] Jiang J. (2007). Linear and Generalized Linear Mixed Models and their Applications. New York, NY: Springer Science & Business Media.

[B16] Kundaje A. Meuleman W. Ernst J. Bilenky M. Yen A. Heravi-Moussavi A.. (2015). Integrative analysis of 111 reference human epigenomes. Nature 518, 317–330. 10.1038/nature14248 25693563 PMC4530010

[B17] Lee S. H. Yang J. Goddard M. E. Visscher P. M. Wray N. R. (2012). Estimation of pleiotropy between complex diseases using single-nucleotide polymorphism-derived genomic relationships and restricted maximum likelihood. Bioinformatics 28, 2540–2542. 10.1093/bioinformatics/bts474 22843982 PMC3463125

[B18] Leslie R. O'Donnell C. J. Johnson A. D. (2014). GRASP: analysis of genotype–phenotype results from 1390 genome-wide association studies and corresponding open access database. Bioinformatics 30, i185–i194. 10.1093/bioinformatics/btu273 24931982 PMC4072913

[B19] Li C. Yang C. Gelernter J. Zhao H. (2014a). Improving genetic risk prediction by leveraging pleiotropy. Hum. Genet. 133, 639–650. 10.1007/s00439-013-1401-5 24337655 PMC3988249

[B20] Li L. Ruau D. J. Patel C. J. Weber S. C. Chen R. Tatonetti N. P.. (2014b). Disease risk factors identified through shared genetic architecture and electronic medical records. Sci. Trans. Med. 6, 234ra57–234ra57. 10.1126/scitranslmed.3007191 24786325 PMC4323098

[B21] Lonsdale J. Thomas J. Salvatore M. Phillips R. Lo E. Shad S.. (2013). The genotype-tissue expression (GTEx) project. Nat. Genet. 45, 580–585. 10.1038/ng.2653 23715323 PMC4010069

[B22] Maier R. Moser G. Chen G.-B. Ripke S. Coryell W. Potash J. B.. (2015). Joint analysis of psychiatric disorders increases accuracy of risk prediction for schizophrenia, bipolar disorder, and major depressive disorder. Am. J. Hum. Genet. 96, 283–294. 10.1016/j.ajhg.2014.12.006 25640677 PMC4320268

[B23] Manolio T. A. Collins F. S. Cox N. J. Goldstein D. B. Hindorff L. A. Hunter D. J.. (2009). Finding the missing heritability of complex diseases. Nature 461, 747–753. 10.1038/nature08494 19812666 PMC2831613

[B24] McCarthy M. I. Abecasis G. R. Cardon L. R. Goldstein D. B. Little J. Ioannidis J. P.. (2008). Genome-wide association studies for complex traits: consensus, uncertainty and challenges. Nat. Rev. Genet. 9, 356–369. 10.1038/nrg2344 18398418

[B25] McCulloch C. E. Searle S. R. Neuhaus J. M. (2008). Generalized Linear and Mixed Models, 2nd Edn. New York, NY: Wiley-Interscience.

[B26] Meyer T. E. Verwoert G. C. Hwang S.-J. Glazer N. L. Smith A. V. van Rooij F. J.. (2010). Genome-wide association studies of serum magnesium, potassium, and sodium concentrations identify six loci influencing serum magnesium levels. PLoS Genet. 6:e1001045. 10.1371/journal.pgen.1001045 20700443 PMC2916845

[B27] Rietveld C. A. Medland S. E. Derringer J. Yang J. Esko T. Martin N. W.. (2013). GWAS of 126,559 individuals identifies genetic variants associated with educational attainment. Science 340, 1467–1471. 10.1126/science.1235488 23722424 PMC3751588

[B28] Ritchie M. D. Holzinger E. R. Li R. Pendergrass S. A. Kim D. (2015). Methods of integrating data to uncover genotype-phenotype interactions. Nat. Rev. Genet. 16, 85–97. 10.1038/nrg3868 25582081

[B29] Sakoda L. C. Jorgenson E. Witte J. S. (2013). Turning of COGS moves forward findings for hormonally mediated cancers. Nat. Genet. 45, 345–348. 10.1038/ng.2587 23535722

[B30] Shin S.-Y. Fauman E. B. Petersen A.-K. Krumsiek J. Santos R. Huang J.. (2014). An atlas of genetic influences on human blood metabolites. Nat. Genet. 46, 543–550. 10.1038/ng.2982 24816252 PMC4064254

[B31] Sivakumaran S. Agakov F. Theodoratou E. Prendergast J. G. Zgaga L. Manolio T.. (2011). Abundant pleiotropy in human complex diseases and traits. Am. J. Hum. Genet. 89, 607–618. 10.1016/j.ajhg.2011.10.004 22077970 PMC3213397

[B32] Smoller J. W. Lunetta K. L. Robins J. (2000). Implications of comorbidity and ascertainment bias for identifying disease genes. Am. J. Med. Genet. 96, 817–822. 10.1002/1096-8628(20001204)96:6<817::AID-AJMG25>3.0.CO;2-A 11121189

[B33] Solovieff N. Cotsapas C. Lee P. H. Purcell S. M. Smoller J. W. (2013). Pleiotropy in complex traits: challenges and strategies. Nat. Rev. Genet. 14, 483–495. 10.1038/nrg3461 23752797 PMC4104202

[B34] Stearns F. W. (2010). One hundred years of pleiotropy: a retrospective. Genetics 186, 767–773. 10.1534/genetics.110.122549 21062962 PMC2975297

[B35] The ENCODE Project Consortium. (2012). An integrated encyclopedia of DNA elements in the human genome. Nature 489, 57–74. 10.1038/nature11247 22955616 PMC3439153

[B36] The International HapMap Consortium. (2005). A haplotype map of the human genome. Nature 437, 1299–1320. 10.1038/nature04226 16255080 PMC1880871

[B37] The Wellcome Trust Case Control Consortium. (2007). Genome-wide association study of 14,000 cases of seven common diseases and 3,000 shared controls. Nature 447, 661–678. 10.1038/nature05911 17554300 PMC2719288

[B38] Uhlén M. Fagerberg L. Hallström B. M. Lindskog C. Oksvold P. Mardinoglu A.. (2015). Tissue-based map of the human proteome. Science 347:1260419. 10.1126/science.1260419 25613900

[B39] Van der Sluis S. Dolan C. V. Li J. Song Y. Sham P. Posthuma D.. (2015). MGAS: a powerful tool for multivariate gene-based genome-wide association analysis. Bioinformatics. 31, 1007–1015. 10.1093/bioinformatics/btu783 25431328 PMC4382905

[B40] Vattikuti S. Guo J. Chow C. C. (2012). Heritability and genetic correlations explained by common SNPs for metabolic syndrome traits. PLoS Genet. 8:e1002637. 10.1371/journal.pgen.1002637 22479213 PMC3315484

[B41] Visscher P. M. Brown M. A. McCarthy M. I. Yang J. (2012). Five years of GWAS discovery. Am. J. Hum. Genet. 90, 7–24. 10.1016/j.ajhg.2011.11.029 22243964 PMC3257326

[B42] Wadhwa R. Song S. Lee J.-S. Yao Y. Wei Q. Ajani J. A. (2013). Gastric cancer-molecular and clinical dimensions. Nat. Rev. Clin. Oncol. 10, 643–655. 10.1038/nrclinonc.2013.170 24061039 PMC3927982

[B43] Welter D. MacArthur J. Morales J. Burdett T. Hall P. Junkins H.. (2014). The NHGRI GWAS Catalog, a curated resource of SNP-trait associations. Nucleic Acids Res. 42, D1001–D1006. 10.1093/nar/gkt1229 24316577 PMC3965119

[B44] Wood A. J. Abernethy D. R. Schwartz J. B. (1999). Calcium-antagonist drugs. New Engl. J. Med. 341, 1447–1457. 10.1056/NEJM199911043411907 10547409

[B45] Wood A. R. Esko T. Yang J. Vedantam S. Pers T. H. Gustafsson S.. (2014). Defining the role of common variation in the genomic and biological architecture of adult human height. Nat. Genet. 46, 1173–1186. 10.1038/ng.3097 25282103 PMC4250049

[B46] Yang C. Li C. Kranzler H. R. Farrer L. A. Zhao H. Gelernter J. (2014). Exploring the genetic architecture of alcohol dependence in African-Americans via analysis of a genomewide set of common variants. Hum. Genet. 133, 617–624. 10.1007/s00439-013-1399-8 24297757 PMC3988209

[B47] Yang J. Benyamin B. McEvoy B. P. Gordon S. Henders A. K. Nyholt D. R.. (2010). Common SNPs explain a large proportion of the heritability for human height. Nat. Genet. 42, 565–569. 10.1038/ng.608 20562875 PMC3232052

[B48] Yang J. Lee S. H. Goddard M. E. Visscher P. M. (2011). GCTA: a tool for genome-wide complex trait analysis. Am. J. Hum. Genet. 88, 76–82. 10.1016/j.ajhg.2010.11.011 21167468 PMC3014363

[B49] Zhou X. Stephens M. (2014). Efficient multivariate linear mixed model algorithms for genome-wide association studies. Nat. Methods 11, 407–409. 10.1038/nmeth.2848 24531419 PMC4211878

[B50] Zhu Q. Wong A. K. Krishnan A. Aure M. R. Tadych A. Zhang R.. (2015a). Targeted exploration and analysis of large cross-platform human transcriptomic compendia. Nat. Methods 12, 211–214. 10.1038/nmeth.3249 25581801 PMC4768301

[B51] Zhu X. Feng T. Tayo B. O. Liang J. Young J. H. Franceschini N.. (2015b). Meta-analysis of correlated traits via summary statistics from GWASs with an application in hypertension. Am. Jx. Hum. Genet. 96, 21–36. 10.1016/j.ajhg.2014.11.011 25500260 PMC4289691

